# Human motion segmentation and recognition using machine vision for mechanical assembly operation

**DOI:** 10.1186/s40064-016-3279-x

**Published:** 2016-09-21

**Authors:** Qiannan Jiang, Mingzhou Liu, Xiaoqiao Wang, Maogen Ge, Ling Lin

**Affiliations:** School of Mechanical and Automotive Engineering, Hefei University of Technology, 193 Tunxi Road, Hefei, 23009 Anhui China

**Keywords:** Motion recognition, Mechanical assembly operation, Key frame extraction, SIFT feature points, Support vector machine

## Abstract

The observation, decomposition and record of motion are usually accomplished through artificial means during the process of motion analysis. This method not only has a heavy workload, its efficiency is also very low. To solve this problem, this paper proposes a novel method to segment and recognize continuous human motion automatically based on machine vision for mechanical assembly operation. First, the content-based dynamic key frame extraction technology was utilized to extract key frames from video stream, and then automatic segmentation of action was implemented. Further, the SIFT feature points of the region of interest (ROIs) were extracted, on the basis of which the characteristic vector of the key frame was derived. The feature vector can be used not only to represent the characteristic of motion, but also to describe the connection between motion and environment. Finally, the classifier is constructed based on support vector machine (SVM) to classify feature vectors, and the type of therblig is identified according to the classification results. Our approach enables robust therblig recognition in challenging situations (such as changing of light intensity, dynamic backgrounds) and allows automatic segmentation of motion sequences. Experimental results demonstrate that our approach achieves recognition rates of 96.00 % on sample video which captured on the assembly line.

## Background

Gilbreth (1917) said that the world’s largest waste is the waste of motion. Therefore, we should find the issues of action and improve workers’ movement through motion analysis, thereby eliminating the waste of time, alleviating fatigue and improving work efficiency (Salvendy 2001; Florea et al. 2003). The first steps of motion analysis are to decompose, identify and record motion sequence, which are performed through the repeated manual observation of operations, in a general way. This is the main reason why motion analysts have the problem of heavy workload and inefficiency. Therefore, in order to reduce the workload and improve the efficiency of motion analysis, a novel method is needed, which could automatically accomplish the motion segmentation, recognition and record by machine.

With the development of image acquisition technology and image processing technology, human motion recognition based on machine vision has become an active area with applications in several domains such as visual surveillance (Lao et al. 2009), video retrieval, patient monitoring (Jalal et al. 2012) and human–computer interaction (Poppe 2010; Turaga et al. 2008). However, segmentation and recognition of continuous human motion for mechanical assembly operation is both one of the most common and most difficult problems in the area of machine vision. Campbell et al. (1996) utilized 3D data gathered in real-time from stereo video cameras and HMMs to learn and recognize gestures. Davis et al. (1997) proposed a view-based approach for the representation and recognition of action and used 18 aerobics exercises to test it. This technique was also incorporated into the Kids Room: an interactive, narrative play-space for children. Carlsson and Sullivan (2001) presented a matching algorithm which matches shape information extracted from individual frames to store prototypes representing key frames of the action to recognize specific actions of tennis players in long video sequence. Stauffer and Grimson (2000) developed a visual monitoring system to track people in indoor environments and outdoor environments using multiple cameras. Laptev (2005) applied space–time features to detect walking people, along with occlusions and dynamic cluttered backgrounds. Ellis et al. (2007) proposed a novel dynamic context model to classify the behavior of group interactions in smart meeting room environment. Niebles et al. (2008) proposed a novel unsupervised learning algorithm using latent topic models to categorize and localize the human actions, and this algorithm was tested on challenging datasets: the KTH human motion dataset, the Weizmann human action dataset, and figure skating actions dataset. Liu et al. (2008) proposed a human actions recognition method using multiple features which was tested on publicly available data sets. Chen et al. (2009) applied MoSIFT algorithm to recognize human actions in surveillance videos. Recognition of human actions in surveillance videos is a part of the TRECVID Event Detection task. Shi et al. (2011) proposed a discriminative semi-Markov model approach to solve the inference problem of simultaneous segmentation and recognition, and this model was verified on KTH dataset. Zhang et al. (2012) proposed a human action recognition approach based on an improved BOW model and latent topic model. Cui et al. (2012) proposed a Matrix-based approach for unsupervised human action categorization. The above two approaches were tested on two datasets, the KTH datasets and WEIZMANN datasets. Jiang et al. (2012a) proposed a shape-motion prototype-based approach, and this approach achieved recognition rates of 92.86 % on a large gesture dataset with dynamic backgrounds. Bousmalis et al. (2013) presented the infinite HCRF (iHCRF), which is capable of automatically learning the optimal number of hidden states for a classification task. The UNBC-McMaster Shoulder Pain Expression database was used for experimental verification. Ellis et al. (2013) presented algorithms for reducing latency of recognizing actions in designing interactive, and evaluated these algorithms on two existing datasets—the MSR Action 3D dataset and the MSRC-12 Kinect Gesture dataset. Reddy and Shah (2013) proposed an action recognition method using the scene context information and motion features to solve the action recognition problem on a HMDB51 dataset. Park and Trivedi (2005) developed a driver activity analysis system using a rule-based decision tree to recognize driver activity. Kim and Medioni (2008) proposed a key visual functionality to recognize six kinds of human actions (walking, sitting, raising hand, lying, falling, and standing up) in the Intelligent Home environment. Cisek et al. (2014) used foreground-weighted histogram decomposition to recognize human action on three datasets:UCF50, HMDB51, and Olympic sports. Slama et al. (2014) developed an accurate action-recognition system using learning on the Grassmann manifold to recognize human action and activity on three public 3D action datasets: MSR-action3D, UT-kinect and UCF-kinect datasets. Yu and Lee (2015) proposed a dynamic classification model called supervised MTRNN for human action classification and the inference of mental states. 16 samples corresponding to each action were used to test this model. Guo and Chen (2015) applied regularized multi-task learning base on spatial–temporal feature to recognize human actions on the TJU dataset. However, the issue of segmentation and recognition of continuous human motion for mechanical assembly operation has not been previously discussed in the literature.

The segmentation and recognition of continuous human motion for mechanical assembly operation is a problem that has challenge sex in the field of machine vision.

1. A continuous video sequence contains a series of motions, and there is no obvious boundary between motions. In addition, the speed of motion affect the motion time. Therefore, unsupervised motion segmentation is quite difficult.
2. Imaging conditions such as light intensity and image background are constantly changing in a realistic assembly environment.
3. There is close connection between human motion and the reality environment, so the types of human motion not only depend on the characteristics of motions, but also is directly related to objects in the environment.

The above three points affect the robustness of the segmentation and recognition algorithm of continuous human motion in the assembly environment. In this paper, we proposed a novel automatic segmentation and recognition method for assembly operations. In order to solve the first problem, the proposed method can segment motion in continuous video using dynamic key frame extraction technology based on content. In order to solve the second problem, we use SIFT feature points matching to find the feature points of ROIs, and the SVM is used to build the classifier to classify the feature vector, which make the recognition of motion possess good robustness. The method also extracts the feature points of the human hand and the assembly work piece, and the displacement vector between the feature points is used to represent the relationship between the human and the environment, and then the third problem is solved.

This paper presents an automated segmentation and recognition method that effectively accomplishes the observation, decomposition and record of human motion using SVM and machine vision in assembly environment. The remainder of this paper is organized as follows. Second section describes key frame extraction and feature points extraction of ROIs. Third section presents the proposed motion recognition algorithm. Fourth section demonstrates the experimental validation process, and conclusions are drawn in the last section.

### Key frames extraction and preprocessing

#### Key frame extraction

In order to reduce the number of the images to be processed and ensure the action recognition algorithm is timely, key frame extraction from the video stream is required before image analysis and recognition. The criterion of key frame extraction is to calculate the dissimilarity between image frames (Chatzigiorgaki and Skodras 2009). Content-based key frame extraction is based on the change of visual information such as color and texture of the image (Lew et al. 2006). If the visual information of a frame varies significantly, the frame is the key frame and is extracted. A novel content-based dynamic key frame extraction algorithm is implemented as follow:

Assume that the video stream contains *S* images, and every frame image has *P*_1_ × *P*_2_ pixels. The gray value of each pixel is represented by $H\left( {a,\;b} \right) \, \left( {a = 0, \, 1, \, 2, \ldots ,P_{1} - 1;\;b = 0, \, 1, \, 2, \, \ldots ,P_{2} - 1} \right)$. Each image is divided into *K*_1_ × *K*_2_ sub-blocks with same size, and every sub-block contains $\bar{P}$ pixels.

1 $$\bar{P} = \frac{{P_{1} \times P_{2} }}{{K_{1} \times K_{2} }}$$

The average gray value of each sub-block is expressed as follows:

2 $$E\left[ {H_{t} } \right] = E\left[ {H_{t} \left( {i,j} \right)} \right] = \frac{1}{{\bar{P}}}\mathop \sum \limits_{k = 1}^{{\bar{P}}} H(a,b)$$

$$\left( {i = 0, \, 1, \, 2, \ldots ,K_{1} - 1;j = 0, \, 1, \, 2, \ldots ,K_{2} - 1;t = 0, \, 1, \, 2, \ldots ,N - 1} \right)$$

where *i* and *j* represent the row of sub-block and the column of sub-block respectively. $E\left[ {H_{t} \left( {i, j} \right)} \right]$ is the average gray value of the sub-block which is in the *i*th row and *j*th column. Therefore, the average gray value $E\left( H \right)$ of each image and the dispersion degree $\sigma^{2} \left( {{\text{E}}\left[ {H_{t} \left( {i, j} \right)} \right]} \right)$ of gray value of every sub-block can be obtained.

3 $$E\left( H \right) = \frac{1}{{K_{1} \times K_{2} }}\mathop \sum \limits_{i = 0}^{{K_{1} - 1}} \mathop \sum \limits_{j = 0}^{{K_{2} - 1}} E\left[ {H_{t} \left( {i,j} \right)} \right]$$

4 $$\sigma^{2} \left( {E\left[ {H_{t} } \right]} \right) = \left\{ {E\left[ {H_{t} \left( {i,j} \right)} \right] - E\left( H \right)} \right\}^{2}$$

The feature vector of any image *S* is expressed as follows:

5 $$F_{s} = \left\{ {E\left[ {H_{1} } \right]^{s} ,\sigma^{2} \left( {E\left[ {H_{1} } \right]} \right)^{s} , \ldots ,E\left[ {H_{N} } \right]^{s} ,\sigma^{2} \left( {E\left[ {H_{N} } \right]} \right)^{s} } \right\}$$

The feature vector of the *p*th image is represented as *F*_*p*_. The feature vector of the *q*th image is represented as *F*_*q*_. Then the Euclidean distance of the two feature vectors is calculated by the following equation:

6 $$Dis\left( {F_{p} ,F_{q} } \right) = sqrt\left\{ {\mathop \sum \limits_{k = 1}^{N} \left( {E\left[ {H_{t} } \right]^{p} - E\left[ {H_{t} } \right]^{q} } \right)^{2} + \left( {\sigma^{2} \left( {E\left[ {H_{t} } \right]} \right)^{p} - \sigma^{2} \left( {E\left[ {H_{t} } \right]} \right)^{q} } \right)^{2} } \right\}$$

**Fig. 1:**
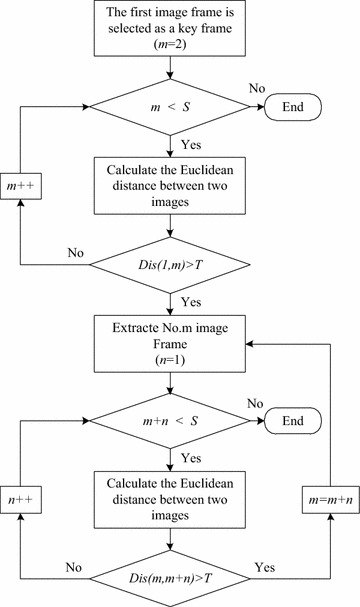
Key frame extraction process

The first frame is set as key frame, which is extracted from video stream, and the value of threshold *T* is calculated. The threshold *T* is a parameter used for determining whether the image is a key frame or not. The Euclidean distance of the two neighboring frame of images is obtained by Inter-frame Difference Algorithm and the set *D* of all Euclidean distances is composed. The set *D* of all Euclidean distances is expressed as follows:

7 $$D = \left\{ {Dis\left( {F_{1} ,F_{2} } \right),Dis\left( {F_{2} ,F_{3} } \right), \cdots ,Dis\left( {F_{m} ,F_{m + 1} } \right), \cdots ,Dis\left( {F_{S - 1} ,F_{S} } \right)} \right\}$$

where the set *D* contains *S*−1 elements. And using the formula (8), the average value $\overline{Dis}$ of all elements is calculated. The value of $\overline{Dis}$ is taken as the value of *T.* The key frame is extracted as shown in Fig. 1.

8 $$\overline{Dis} = \frac{{\sum\nolimits_{m = 1}^{S - 1} {Dis\left( {F_{m} ,F_{m + 1} } \right)} }}{S - 1}$$

Firstly, calculate the Euclidean distance between the feature vectors of the first key frame and following frames by function (6). If the distance is over *T*, the subsequent *m* frame is the key frame, which is extracted from the video stream. Extract key frames from video stream as described in the previous steps until the last frame is calculated. Following the above steps, we can get a key frame sequence of the video stream finally.

#### ROIs extraction of hand

**Fig. 2:**
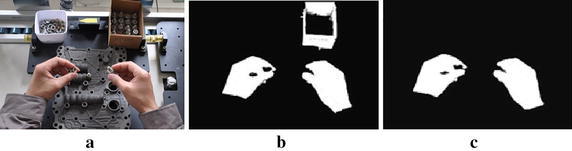
The process of hand detection. **a** Original hand image. **b** Skin color regions. **c** Hand regions

Complexion is one of the most significant features of hand. Complexion has a favorable invariance for the image scaling, translation and rotation. It also has certain robustness to the change of image dimensional, capturing direction and angle. (Van den Bergh and Van Gool 2011; Kurakin et al. 2012). Therefore, by use of complexion information is the most effective and direct way to detect human hand. Range of skin color is much more compact and hand extraction is less susceptible to the effects of light and other objects in YCbCr and HSV space than in RGB space. But the color space transformation between RGB and HSV is more complicated than that between RGB and YCbCr, so human hand complexion is modeled and segmented in YCbCr space. First of all, the RGB color space is transformed into the YCbCr color space, threshold segmentation is made to the Cr component. Then, we segment the skin color regions with complex backgrounds based on skin color model. After segmentation is finished, multiple connected domains are obtained. The empty of the connected domains are filled by closing operation of mathematical morphology. Finally, the hand objects can be identified according to the hand shape feature. This process of hand detection is shown in Fig. 2.

#### ROIs extraction of workpiece

Template matching algorithm is widely used in projects as a classic recognition algorithm. Input template and target templates are compared to decide whether the two templates are the same. Using the results of template matching to determine whether an image contains target templates and find the locations of target templates in the image (Jain and Zongker 1997). The advantage of template matching is lesser amount of calculation, and the disadvantage is that the robustness of template matching is poor and recognition result is directly dependent on template construction (Pereira and Pun 2000). Because the shape characteristic of workpiece is really good, the shape-based template matching algorithm is adopted to detect the ROIs of workpiece. When serious block, confusion and nonlinear illumination changes happen on image, the algorithm still has a very high recognition rate (Mohan et al. 2001; Breuer et al. 2007). The algorithm always uses the inner product of the gradient vector of pixel as similarity measure. The best matching location is searched by calculating the minimum inner product. At the same time, In order to speed up the searching process, the pyramid of the multi-level images was built. Pyramid image-matching strategy is adopted to realize fast ROI extracting (Tanimoto 1981).

#### Feature points extraction of ROI

Lowe (2004) proposed an optimized SIFT characteristic operator. The operator is invariant to luminance, perspective, translation, rotation and scale change. It also maintains a better matching result, despite the external factors such as shape and background change, environmental noise, and occlusion. Thus, the matching algorithm based on SIFT descriptor has been successfully applied in object recognition, robot location, fingerprint and face recognition and other fields (Mortensen et al. 2005; Li et al. 2010; Mikolajczyk and Schmid 2005).

The SIFT feature points extraction and matching algorithm includes three steps as follow:

- Detect SIFT feature points of ROIs from the sample image and key frame image
  SIFT feature point detection is essentially local extreme point detection in different Gaussian (DOG) scale spaces. Each pixel point of ROI is compared with its 26 neighborhood points which contain eight adjacent points in the same scale space and eighteen points in vertically adjacent scale spaces, if a point is a maximum or minimum, the point is the feature point of ROI in the scale (Lowe 2004). Because the DOG value is sensitive to noise and edge, local extreme points need to be further checked for determination of feature points (Aprovitola and Gallo 2014).
- Generate SIFT feature point descriptor
  Firstly, samples were collected from the neighborhood window of feature points and were subjected to gradient histogram calculating. The peak position of the histogram is the main direction of the gradient of the feature points. The feature point’s main direction makes the feature points possess rotational invariance (May et al. 2012). In order to improve the stability of matching, 128-dimensional vector is use to describe SIFT feature point descriptor.
- Obtain SIFT feature points set of key frame using feature points matching
  The Euclidean distance between SIFT feature points of sample image and SIFT feature points of key frame is calculated, and its value is used as the similarity measure between feature points. Through the above mentioned calculation, nearest neighbor and second nearest neighbor of the SIFT feature point of sample image can be found from all SIFT feature points in the key frame. The ratio of closest distance A and second closest distance B is $d\left( {a,b} \right)$. If $d\left( {a,b} \right)$ is less than *T*_*a*,*b*_, the SIFT feature point of template image and the SIFT feature point of key frame are matched (Jiang et al. 2012b). By use of the above method, the SIFT feature points set of ROI in key frame is obtained through feature points matching.

### Proposed motion recognition algorithm

**Fig. 3:**
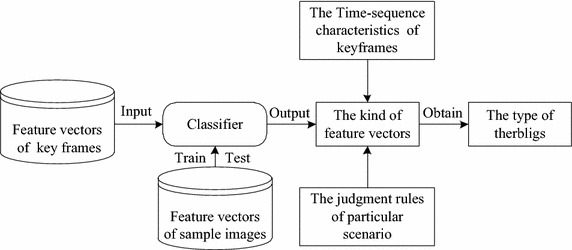
Algorithm process of motion recognition

First of all, the multiple displacement vector sets can be obtained by calculating the displacement between feature points of different ROIs, and the displacement vector sets are the feature vectors of key frame. The feature vector classifier should be obtained by training and testing before motion recognition. Eventually, input the feature vectors of key frames into the classifier to identify the kind of feature vector, and identify the type of therblig based on the time-sequence characteristics of key frames and the judgment rules of particular scenario. Motion recognition algorithm flow is illustrated as shown in Fig. 3.

#### Get feature vectors from key frame

The ROIs of image consist of human hands and two workpieces in mechanical product assembly process. The assembling process needs to use both hands to accomplish a task. Left hand’s feature points set *M*_1_ (contains *m*_1_ feature points), right hand’s feature points set *M*_2_ (contains *m*_2_ feature points), workpiece 1 feature points set *N*_1_ (contains *n*_1_ feature points) and workpiece two feature points set *N*_2_ (contains *n*_2_ feature points) can be obtained through the SIFT feature point matching. If the displacement vectors set between feature points of points set *A* and feature points of points set *B* is denoted by (*A*, *B*), we can get six displacement vectors sets (*M*_1_, *M*_2_), (*M*_1_, *N*_1_), (*M*_1_, *N*_2_), (*M*_2_, *N*_1_), (*M*_2_, *N*_2_) and (*N*_1_, *N*_2_) by any combination of all four feature points sets, and feature points of hands are the starting points of the displacement vector. The above six displacement vectors sets constitute the feature vectors of key frame, and each kind of displacement vectors set includes *R*_*i*_ (*i* = 1, 2 , 3, 4, 5, 6) displacement vectors. *R*_*i*_ satisfies the following equation:

9 $$R_{i} = A^{j} \times B^{k}$$

where *A*^*j*^ is the number of feature points in points set *A* and *B*^*k*^ is the number of feature points in points set *B*.

#### Construct classifier based on SVM

SVM is a kind of novel machine learning method which developed on the basis of statistical learning theory. Compared with traditional learning method, SVM employs structural risk minimization criterion to minimize the learning error and simultaneously decrease the generalization error. It has been developed for solving the classification and regression problems, and it also is a major achievement in machine learning research in recent years (Vapnik 2000). There are many unique advantages of SVM in solving small samples, nonlinear and high-dimensional pattern recognition problems (Brezak et al. 2012). It can not only find the global optimal solution from the limited sample information, but also describe the train sample accurately and identify any test samples without error (Vapnik 2000). For the method based on back propagation (BP) neural network or Radial basis function (RBF) neural network, when the dimension of input vector is more, it may cause the network scale is too large, which leads to difficulty in training and other issues. While the computations load of the SVM method is almost independent of input vector dimension, therefore, it is suitable to deal with the problem of large input dimension. In recent years, SVM-based method had been widely applied in texture classification, time series forecast and face recognition because it need much less training time and carry out an efficient calculation (Benkedjouh et al. 2015; Kao et al. 2014; He and Li 2014). Therefore, SVM will be used as a classification algorithm of motion recognition method in this paper.

**Fig. 4:**
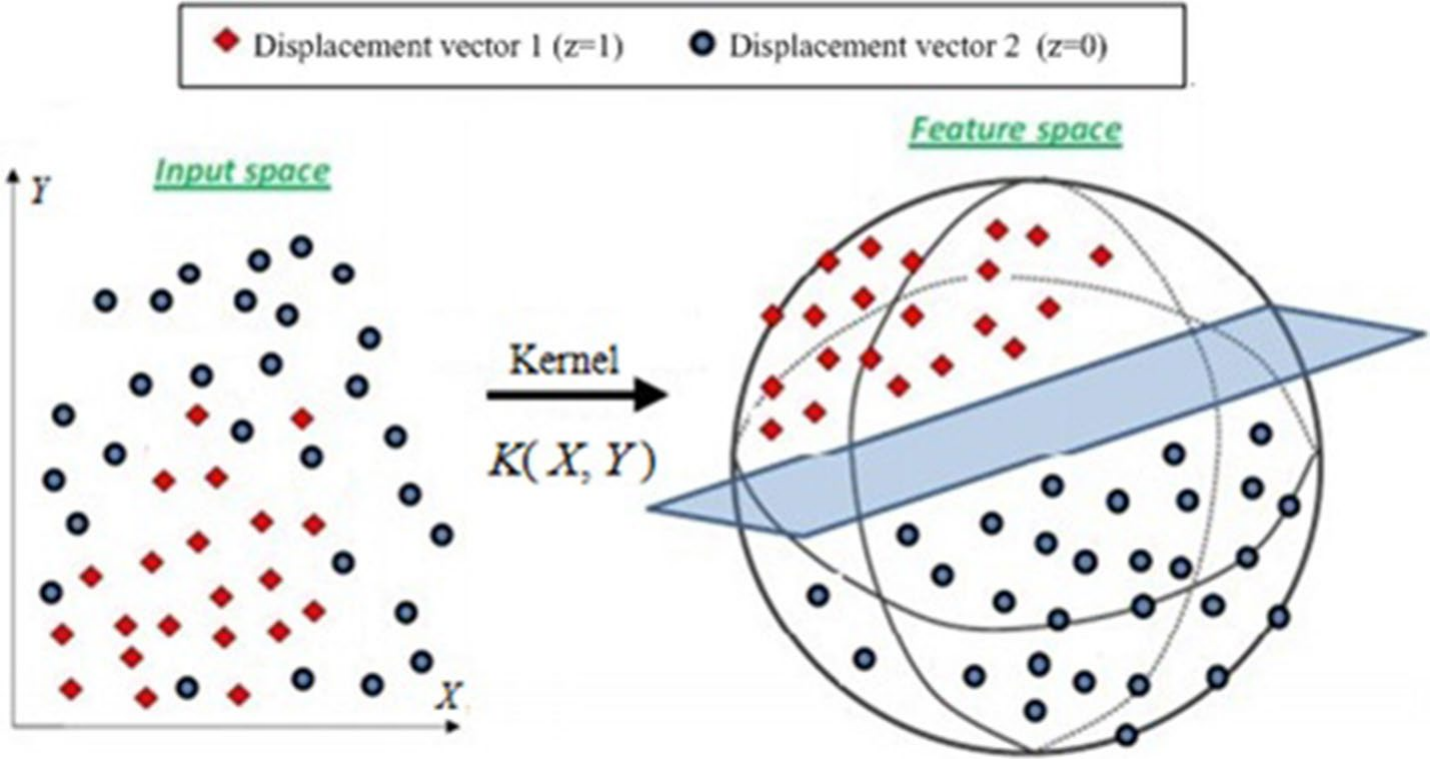
The mapping process *Displacement vector 1* indicates that two ROIs are close to each other; *Displacement vector 2* indicates that two ROIs are not close to each other; Input space is the low dimensional space; Feature space is the high dimensional space which determined by kernel functions

The process of classifier construction based on SVM is as follows:

- Sample preparation
  Sample images were screened out from video stream, and the pictures are handled as the above- described way, then the displacement vectors set between ROIs can be obtained, which is a feature vector of image. The type of feature vector is obtained through expert evaluating method before sample training. If two ROIs are close to each other, the type of feature vector is labeled as *z* = {1}; If two ROIs are not close to each other, the type of feature vector is labeled as *z* = {0}. A feature vector is expressed as $\varvec{T} = \left( {A,B} \right) = \left\{ {(x_{1} ,y_{1} ),(x_{2} ,y_{2} ), \ldots ,(x_{k} ,y_{k} ), \ldots ,(x_{K} ,y_{K} )} \right\}\; \left( {k = 1, \, 2, \ldots ,K} \right)$, where (*x*_*k*_, *y*_*k*_) represents the coordinate of each displacement vector and *K* is the number of displacement vectors. Therefore, (***T***, *z*) is regarded as input sample for the training of SVM classifier.
- Classifier construction
  On the basis of the distribution of displacement vectors in a two-dimensional coordinate system, it is shown that the problem is linearly inseparable. To solve the nonlinear problem, the calculation is completed in the low dimensional space firstly. Then SVM maps data from sampling space to higher dimensional characteristic space by kernel functions that satisfy Mercer condition. Eventually, the optimal separating hyperplane is constructed in the high dimensional feature space. Minimize the distance between all sample points and hyperplane, so that the nonlinear problem is converted into linear divisible problem to get optimum relation (Boser et al. 1996; Cristianini and Shawe-Taylor 2000). We map the training samples into a high-dimensional feature space via a nonlinear mapping determined by a kernel function. The mapping process is shown in Fig. 4.

If $\varvec{X} = \varvec{T} = \left( {A,B} \right) = \left\{ {(x_{1} ,y_{1} ),(x_{2} ,y_{2} ), \ldots ,(x_{k} ,y_{k} ), \ldots ,(x_{K} ,y_{K} )} \right\},\;\left( {k = 1, \, 2, \ldots ,K} \right),Y_{k} = z,$ samples set is expressed as $\left\{ {(\varvec{X}_{1} ,Y_{1} ),(\varvec{X}_{2} ,Y_{2} ), \ldots ,(\varvec{X}_{k} ,Y_{k} ), \ldots ,(\varvec{X}_{K} ,Y_{K} )} \right\}$ containing *K* samples, where $\varvec{X}_{k}$ is the input samples and $\varvec{X}_{k} \in R^{2}$, and *Y*_*k*_ (*Y*_*k*_ = 0 or *Y*_*k*_ = 1) is the expect output of the sample data. Map the sample points in two-dimension space to the feature space, and then the optimal classification discriminant function *F*(*X*) is obtained according to the SVM learning algorithm. *F*(*X*) is the feature vector classifier.

10 $$F\left( X \right) = {\text{sgn}}\left(\mathop \sum \limits_{k = 1}^{K} \alpha_{k}^{*} Y_{k} \cdot K\left( {\varvec{X},\varvec{X}_{k} } \right) + b^{*} \right)$$

#### Motion recognition

**Fig. 5:**
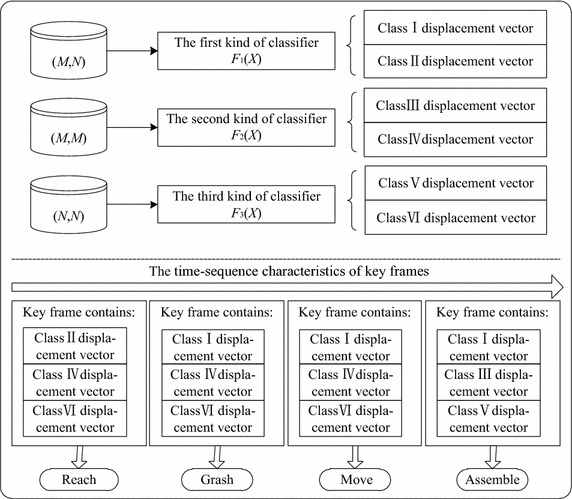
The process of therblig recognition. *Class I displacement vector* indicate that hand is close to workpiece; *Class III displacement vector* indicate that hands are close to each other; *Class V displacement vector* indicate that workpieces are close to each other. Other kinds of displacement vectors indicate that ROIs are not close

Motion recognition is essential to identify the type of therblig in mechanical product assembly process. A simple assembly operation normally includes four types of therbligs: Reach, Grasp, Move and Assemble. Displacement vector can be divided into three kinds for simple mechanical product assembly process: displacement vector (*M*, *N*) of hands relative to workpieces, displacement vector (*M*, *M)* between hands and displacement vector (*N*, *N*) between workpieces. Three types of classifiers *F*_1_ (*X*), *F*_2_ (*X*) and *F*_3_ (*X*) are trained based on the above displacement vectors. Then, the type of therblig in key frames can be recognized by the process of Fig. 5.

First of all, input different kinds of displacement vectors into different classifiers to identify the kind of the displacement vector. Then, identify the type of therblig based on the time-sequence characteristics of key frames and the judgment rules of particular scenario.

- If the key frame contains class II displacement vector, class IV displacement vector and class VI displacement vector, the type of therblig is Reach.
- If the key frame contains class I displacement vector, class IV displacement vector and t class VI displacement vector, the type of therblig is Grasp or Move.
- If the key frame contains class I displacement vector, class III displacement vector and class V displacement vector, the type of therblig is Assemble.

There is much different visual information between the image of containing Grasp and the image of containing Move, so both of these images are key frames and are extracted from video streaming. Since the images of video stream are arranged in chronological order, the key frame of containing Grasp is before the key frame which contains Move. Therefore, Grasp and Move can be separated in accordance with the time-sequence characteristics of key frames.

### Implementation

**Fig. 6:**
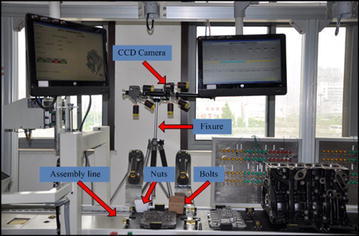
Prototype system in the mechanical product assembly line

In this section, an experiment simulated the real bolt assembly operation of mechanical product. It was provided to verify the feasibility and robustness of the vision-based motion recognition method. The experimental framework consists of an operator, workpieces and an image acquisition system. The workpieces contain bolts (M8 × 15) and hex nuts (M8 × 1.5). The image acquisition system contains the light source, a CCD color camera, a computer and a prototype system in the mechanical product assembly line, as shown in Fig. 6. The light source is a kind of artificial daylight (DH, LER2-90SW2). An industrial CCD progressive scan RGB color camera (DH, SV2000GM/C 1/1.8″ 1628 × 1236, active pixels) mounted with a 16 mm lens (DH, model M3Z1228C-MP) was applied to capture the motion images of operator. The motion images were transmitted from the camera to computer through the Gigabit Ethernet. The HALCON software was used for picture processing and the MATLAB software was used for model analysis in the experiment, and the configuration of the computer is listed as follows: OS: Windows 7(32 bit); CPU: Intel Core i5-3337U; RAM: 6 GB; MATLAB version: 2011b; HALCON version: 11.0. In the actual environment, the background and light intensity are both changed.

Experimental procedure:

- A sample image of left hand was captured by using machine vision system, and the left hand was in a state of grabbing bolt (This example picture is named L-G-P);
- A sample image of right hand was captured by using machine vision system, and the right hand was in a state of grabbing (This example picture is named R-G-P);
- A sample image of hands and the hands was captured by using machine vision system, and the hands were in a state of mounting bolts (This example picture is named D-A-P);
- The video of bolt assembly operations was captured by the machine vision system, and the operator worked with both hands. The video stream contains 500 motion cycles, and each cycle contains four types of therbligs: Reach, Grasp, Move and Assemble.

#### Image preprocessing

Sample images and the image in video stream need a series of preprocessing before the feature extraction. Firstly, the impacts of noise and light were eliminated by use of Gaussian filter (Zhang and Parker 2011; Wu et al. 2012), and the RGB color space was converted to the YCbCr color space. Then the color threshold range was set from 140 to 160 in the Cr component, multiple connected regions were obtained. Then the empty areas of connected regions were filled by closing operation. Eventually, the connected regions larger than 35,000 pixels were extracted. The connected regions were the ROI of hand. After obtaining the ROIs of hand, images were matched to bolt template and nut template; the ROIs of workpiece were obtained.

#### Motion recognition

**Fig. 7:**
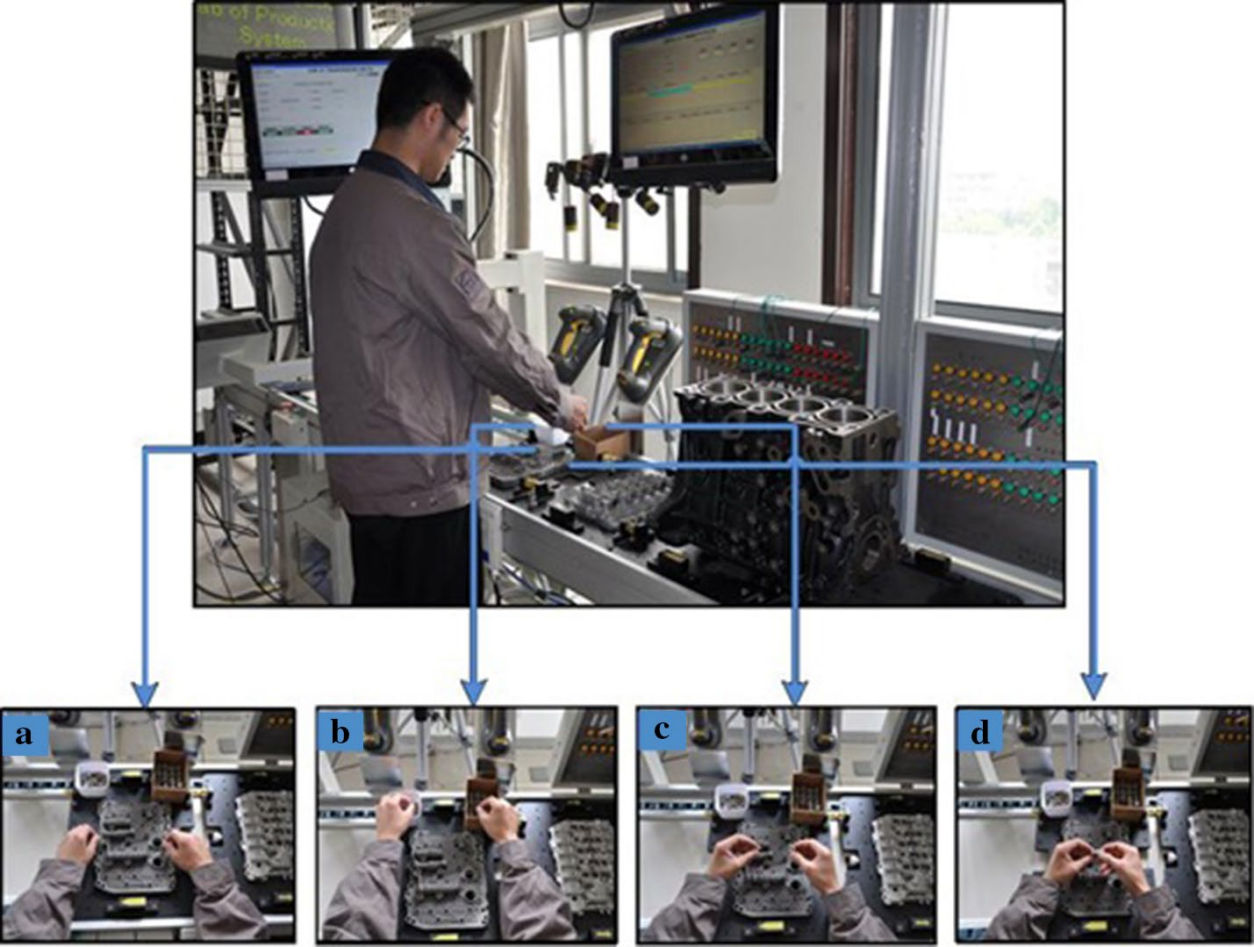
Four key frames

The ROIs of sample images were obtained after preprocessing, then the SIFT feature points of ROIs were detected and SIFT feature point descriptor was generated. Key frames were extracted by the content-based dynamic key frame extraction algorithm. Four examples of key frames were shown as Fig. 7. The as seen in Fig. 7, the technology of video key-frame extraction not only realized key frame extraction but also segmented motion sequences automatically. The feature points of ROIs in key frames were obtained based on the SIFT feature points of sample images, and the feature vectors of key frames were acquired by calculating the displacement vector between feature point sets. Hands were used as starting points to calculate the displacement vector between hand and workpiece; left-hand was taken as starting point to calculate the displacement vector between hands; hex bolt was used as starting point to calculate the displacement vector between workpieces. According to the coordinates of feature points and the calculation rules in the above, the feature vectors of key frames were obtained. The feature vector of each key frame contains six displacement vector sets. The six displacement vector sets were respectively expressed as (*M*_1_, *M*_2_), (*M*_1_, *N*_1_), (*M*_1_, *N*_2_), (*M*_1_, *N*_1_), (*M*_2_, *N*_2_) and (*N*_1_, *N*_2_). The set (*M*_1_, *M*_2_) contains 3364 displacement vectors. The set (*N*_1_, *N*_2_) contains 441 displacement vectors. Each of the four remaining sets of feature vector contains 1218 displacement vectors.

When using the content-based dynamic key frame extraction algorithm for key frame extraction, parameters including *K*_1_, *K*_2_ and *T* must be determined in advance. In this study, the optimal values of the three parameters were determined as *K*_1_ = 16, *K*_2_ = 12, *T* = 14.14. 2524 key frames were extracted from the video stream which contains 500 motion cycles. Before classifier training and data testing, the experts identified the therblig type of each key frame. In this paper, the SVM model was constructed by using Gauss kernel function (Cherkassky and Ma 2004).

11 $$K\left( {X,X_{k} } \right) = \exp \left( { - \frac{{\left| {X - X_{k} } \right|^{2} }}{{2\delta^{2} }}} \right) = \exp ( - \gamma \left| {X - X_{k} } \right|^{2} )$$

**Fig. 8:**
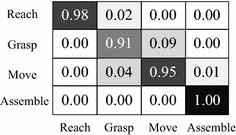
Confusion matrix of motion categories, with an average performance of 96 %. *Rows* represent actual motion categories; *Columns* represent recognition results of our method

We used cross validation method to select the most accurate parameters (*C*, *γ*) as the parameter of classifier model. Among these key frames, 1516 key frames (containing 300 motion cycles) were used for training and 1008 key frames (containing 200 motion cycles) were used for the testing. Each key frame of the above contained three classes of displacement vectors: (*M*, *N*), (*M*, *M*) and (*N*, *N*) which were used to select the parameters of *F*_1_ (*X*), *F*_2_ (*X*) and *F*_3_ (*X*) respectively. The optimal parameters of classifier models were obtained by cross validation: *C*_1_ = 2.0, *γ*_1_ = 0.03125; *C*_2_ = 3.5*, γ*_2_ = 0.05556; *C*_3_ = 5.0*, γ*_3_ = 0.125. Finally, all the 2524 samples were used for final validation. The result confusion matrix is shown in Fig. 8, with an average performance of 96 %.

According to the confusion matrix shown in Fig. 8, we can see that Grasp and Move are more easily to be confused, which are consistent with our observations. Actually some Grasp and Move instances are even difficult to differentiate for people. Grasp and Move do have similar feature vector, but the spatial–temporal characteristic is slightly different. Unfortunately, the spatial–temporal characteristics are difficult to be reflected from the pixel-wise. The experimental result on motion recognition demonstrates the robustness of the proposed method.

In order to demonstrate the advantage of the proposed methods, we compare the results with other reported ones, which are shown in Table 1. The five compared state-of-the-art methods require a pre-segmentation of the continuous motion sequence into elementary segments, a tedious manual operation. We can see that the result of our method is in parallel with the best supervised method (Lu et al. 2015), which recognized human motions by two-level Beta process hidden Markov model, and gain 2.6 % improvement from Reddy and Shah (2013), which used Sphere/Rectangle-tree to construct a novel framework for motion categorization.

**Table 1:** Comparison of different methods on the video of bolt assembly operations

Methods	Recognition objects	Accuracy (%)
*Our method*	Continuous motion sequence	96.00
Schuldt et al. (2004)	Motion segments	71.75
Niebles et al. (2008)	Motion segments	83.30
Jiang et al. (2012a)	Motion segments	84.35
Reddy and Shah (2013)	Motion segments	93.40
Lu et al. (2015)	Motion segments	95.05

#### Record of motion

**Fig. 9:**
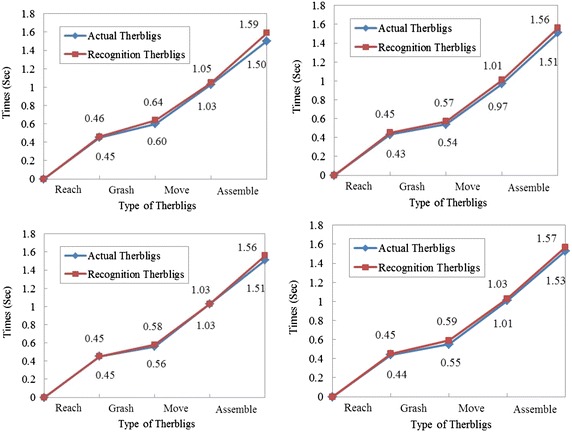
The comparison results of the first four cycles. The actual therbligs were obtained by the experts through the observation, decomposition and recording of motion. The recognition therbligs were the results obtained with such method in this paper

The long video has 500 motion cycles,and each cycle contains four types of therbligs. So there is 2000 therbligs in the video stream. The above study has demonstrated that 2524 key frames were extracted from the video stream. It is clear that the number of key frames is larger than the number of therbligs. Therefore, the adjacent key frames in the video may belong to the same type of therbligs. In order to avoid repeated recording of motion, when the motion types of adjacent key frames are different, the all motions are recorded, else, we record one motion. The results of therblig recognition were compared with actual therbligs, and the comparison results of the first four cycles are as shown in Fig. 9.

Figure 9 shows that the vision-based motion recognition method can accurately identify the type of therbligs, but there are minor differences between the motion times obtained by using the proposed method and observed values. The cause of this phenomenon is that the identification object of the vision-based motion recognition method proposed in this paper is key frame, but not each frame of video stream. If two key frames A and B contain different therbligs, the time interval between them is the time of the therblig in key frame A. When each frame of video stream is used to recognize, the time difference in Fig. 8 would not exist. However, identifying all the frames one by one will greatly reduce timeliness and generate a large amount of redundant data during the recording. Therefore, when the time accuracy of motion analysis is not high, it is reasonable to use the key frame as the object of motion recognition.

## Conclusions

This study proposed a novel machine-vision-based motion segmentation and recognition method for mechanical product assembly operation. The experiment results have demonstrated that the proposed method can segment motion automatically, identify the type of the motion accurately and record each motion and its time. The study makes the following major contributions. (1) The relationship between motion and objects were established by using the displacement vector between SIFT points in different ROIs; (2) In order to improve the timeliness of the proposed method, the key frame extraction technology was applied to reduce the number of images to be processed, and the image processing technique was applied to reduce the number of pixels to be processed; (3) The proposed motion recognition algorithm was constructed based on SVM to classify feature vectors; (4) This method not only accomplishes the motion segmentation, recognition and record automatically but also reduces the workload of motion analysts and improves the efficiency of motion analysis. In addition, the proposed algorithm is general, which can be straightforwardly extended to other motion recognition fields.

## Abbreviations

SVM: support vector machine
ROIs: regions of interest
SIFT: scale invariant feature transform
DOG: difference of gaussian
BP: back propagation
RBF: radial basis function
